# Thermococcus Eurythermalis Endonuclease IV Can Cleave Various Apurinic/Apyrimidinic Site Analogues in ssDNA and dsDNA

**DOI:** 10.3390/ijms20010069

**Published:** 2018-12-24

**Authors:** Wei-Wei Wang, Huan Zhou, Juan-Juan Xie, Gang-Shun Yi, Jian-Hua He, Feng-Ping Wang, Xiang Xiao, Xi-Peng Liu

**Affiliations:** 1 State Key Laboratory of Microbial Metabolism, School of Life Sciences and Biotechnology, Shanghai Jiao Tong University, 800 Dong-Chuan Road, Shanghai 200240, China; www1037554814@sjtu.edu.cn (W.-W.W.); purplexjj@163.com (J.-J.X.); 13166228531@163.com (G.-S.Y.); fengpingw@sjtu.edu.cn (F.-P.W.); zjxiao2018@sjtu.edu.cn (X.X.); 2 Shanghai Institute of Applied Physics, Chinese Academy of Sciences, No. 239 Zhangheng Road, Shanghai 201204, China; zhouhuan@sinap.ac.cn (H.Z.); hejianhua@sinap.ac.cn (J.-H.H.); 3 State Key Laboratory of Ocean Engineering, School of Naval Architecture, Ocean and Civil Engineering, Shanghai Jiao Tong University, 800 Dong-Chuan Road, Shanghai 200240, China

**Keywords:** *Thermococcus eurythermalis*, endonuclease IV, AP site analogue, spacer, DNA repair

## Abstract

Endonuclease IV (EndoIV) is a DNA damage-specific endonuclease that mainly hydrolyzes the phosphodiester bond located at 5′ of an apurinic/apyrimidinic (AP) site in DNA. EndoIV also possesses 3′-exonuclease activity for removing 3′-blocking groups and normal nucleotides. Here, we report that *Thermococcus eurythermalis* EndoIV (TeuendoIV) shows AP endonuclease and 3′-exonuclease activities. The effect of AP site structures, positions and clustered patterns on the activity was characterized. The AP endonuclease activity of TeuendoIV can incise DNA 5′ to various AP site analogues, including the alkane chain Spacer and polyethylene glycol Spacer. However, the short Spacer C2 strongly inhibits the AP endonuclease activity. The kinetic parameters also support its preference to various AP site analogues. In addition, the efficient cleavage at AP sites requires ≥2 normal nucleotides existing at the 5′-terminus. The 3′-exonuclease activity of TeuendoIV can remove one or more consecutive AP sites at the 3′-terminus. Mutations on the residues for substrate recognition show that binding AP site-containing or complementary strand plays a key role for the hydrolysis of phosphodiester bonds. Our results provide a comprehensive biochemical characterization of the cleavage/removal of AP site analogues and some insight for repairing AP sites in hyperthermophile cells.

## 1. Introduction

Both physical and chemical factors in the cell and the environment can cause various types of DNA damage, which will cause some potential mutagenic and toxic effects on the cell. This DNA damage mainly include hydrolytic deamination of cytosine, methylation of bases, oxidized bases, base losses, base crosslinking, DNA strand breaks, and misincorporation in DNA replication [1,2,3]. Among these types of DNA damage, base losses, also called apurinic/apyrimidinic (AP) sites, are the most common lesion [2]. AP sites mainly result from spontaneous base loss via depurination/depyrimidination, as well as removing damaged bases by all kinds of DNA glycosylases [4]. Depurination/depyrimidination creates 2000–10,000 AP sites per cell per day in mammalian cells [5]. AP sites cannot guide the incorporation of a correct (d) NMP because of the inability to form the required hydrogen bonds, and then it will inhibit replication or transcription. When the DNA polymerase encounters an AP site on the template strand, generally dAMP is preferred for incorporation into the extending strand, i.e., the “A-rule” [6]. Therefore, if AP sites are not quickly repaired, the cells will be in danger of serious toxicity.

In the cell, AP sites are mainly repaired by the base excision repair (BER) pathway. BER is initiated by DNA glycosylases, which generate the immediate product AP site [4]. In BER, the enzymes responsible for cleaving the DNA backbone at AP sites are classified into two groups, the AP lyase and the AP endonuclease. The AP lyases cleave the AP site (deoxyribose) through a β-elimination reaction, generating 5′-phosphate and 3′-α,β unsaturated aldehyde [7]. The AP endonucleases, including endonuclease IV (EndoIV) and exonuclease III (ExoIII), incise DNA 5′ to AP sites through hydrolysis of the phosphodiester bond to yield a free 3′-OH and a 5′-deoxyribose-phosphate (dRP) group [8]. In addition to the natural AP site, the C1′-reduced (tetrahydrofuranyl) and deoxyribose-oxidized sites (C1′-oxidized 2-deoxyribonolactone and the C4′-oxidized AP site) are also recognized by AP endonuclease [9,10]. In addition to AP endonuclease activity, EndoIV and ExoIII have 3′-exonuclease activity, and function as 3′-repair diesterases by removing DNA 3′-blocking groups such as 3′-phosphates, 3′-phosphoglycolates and 3′-α,β unsaturated aldehydes [11,12]. On cleaving the DNA 5′ to an AP site or removing the 3′-blocking groups, DNA polymerase will resynthesize a matched DNA strand by incorporating correct dNMPs into the 3′-OH end under the direction of a complementary template strand, and then DNA ligase will seal the nick generated in the repair process [8].

Though both EndoIV and ExoIII primarily function as an AP endonuclease and a 3′-repair diesterase, they have many contrasting properties. ExoIII has strong 3′-exonuclease activity on double-stranded (ds) DNA and endonuclease activity at urea damage site in DNA [13,14]. Bacterial ExoIII also possesses a strong ribonuclease H activity [11]. Human APE1, the homologue of bacterial ExoIII, has several novel activities, such as endoribonuclease [15,16], 3′-RNA phosphatase and 3′-exoribonuclease activities [17]. In addition to the weaker 3′-exonuclease activity on normal 3′-nucleotides [18,19], EndoIV has an additional activity that cleaves DNA 5′ to some oxidative bases [20,21,22]. ExoIII is constitutively expressed in *Escherichia coli* (*E*. *coli*); however, EndoIV is induced by oxidative stress [23]. Human cells use an ExoIII homolog APE1 for treating AP sites [24]; however, *Saccharomyces cerevisiae* (*S. cerevisiae*) uses a homolog of EndoIV, APN-1, for repairing AP sites [25].

The hydrolysis mechanism of the AP site has been interpreted based on the crystal structures of bacterial *E. coli* EndoIV (EcoendoIV) [26,27,28], *Thermus thermophilus* (*T. thermophilus*) EndoIV [29], human APE1 complexed with AP-site-containing double-stranded DNA (dsDNA) [30,31], and bacterial ExoIII from *E. coli* [32]. The recognition of the AP site and subsequent hydrolysis of the phosphodiester bond are involved in the interaction between EcoendoIV and two strands of DNA duplex [26]. Human APE1 interacts with 9–10 nucleotides around the AP site, mainly through weak additive contacts with phosphate groups [30]. The crystal structure of *E. coli* ExoIII gives a detailed interpretation on the catalytic mechanism of the AP endonuclease activity [32]. A crystal structure of ExoIII from a hyperthermophilic archaea, *Archaeoglobus fulgidus*, was also solved [33]. However, until now, no crystal structure of archaeal EndoIV has been solved.

High temperature results in more DNA damage, such as hydrolytic deamination of cytosine and AP sites [5,34], so hyperthermophiles face a serious high-temperature threat to genome integrity and bear more stress to repair DNA damages than mesophiles. *Thermococcus eurythermalis* (*T. eurythermalis*) *A501* is a conditional piezophilic hyperthermophilic archaea, isolated from the Guaymas Basin, that is well adapted to the hydrothermal environment [35]. Except for the EndoIV from *Pyrococcus furiosus* [36], reports on archaeal EndoIV are scarce. *P. furiosus* EndoIV (PfuendoIV) possesses both AP endonuclease and 3′ exonuclease activities, and its 3′-exonuclease activity, but not its AP endonuclease activity, is stimulated by PCNA [36]. Meanwhile, the effects of the structure and context of AP site analogues on EndoIV activity are less known. *T. eurythermalis* encodes a homologue of EndoIV that shows very low sequence similarity to EcoendoIV. As the only AP endonuclease, *T. eurythermalis* EndoIV (TeuendoIV) might play important roles in repairing DNA damage related to AP sites. To understand the enzymatic properties of EndoIV from hyperthermophiles, we biochemically characterized the cleavage reaction of TeuendoIV using the DNAs containing various analogues as substrates. The AP endonuclease activity of TeuendoIV can hydrolyze the phosphodiester bond 5′ to various AP site analogues, including the polyethylene glycol Spacer and alkane Spacer. For Spacers longer than three atoms, the cleavage reaction is highly efficient, and the shorter Spacer C2 strongly inhibits the cleavage reaction. However, the efficient cleavage of a Spacer adjacent to the 5′-terminus requires at least two normal nucleotides located at the 5′-end. In addition, the 3′-repair diesterase activity of this enzyme can remove one or more consecutive AP sites at the 3′-terminus. Finally, we confirmed that the residues that interact with the bases or phosphate-deoxyribose backbone around the AP site are most important for hydrolyzing the phosphodiester bond 5′ to AP sites. Our results provide biochemical information on repairing AP sites in hyperthermophilic archaea.

## 2. Results

### 2.1. TeuendoIV Possesses AP (Apurinic/Apyrimidinic) Endonuclease Activity

**Figure 1:**
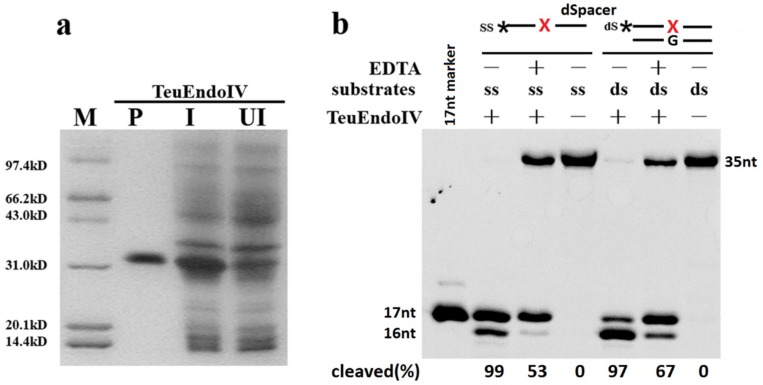
AP endonuclease activity of TeuendoIV. (**a**) 15% SDS-PAGE analysis of recombinant TeuendoIV. Lane M, molecular weight marker; lane P, purified recombinant TeuendoIV; lanes I and UI denote induced and uninduced *E. coli* total proteins. (**b**) Cleavage of ssDNA and dsDNA carrying a synthetic AP site, dSpacer, by TeuendoIV. The reaction mixtures contained 20 mM Tris-HCl pH 7.6, 100 mM NaCl, 100 nM AP site-containing dsDNA (AP/G) or ssDNA, and 5 nM TeuendoIV and were incubated at 55 °C for 10 min. EDTA (2 mM) was included or not. A 17-nt ssDNA was loaded onto the gel as marker to confirm the product. The symbol of black asterisk and the red letter X denote the fluorescein (6-FAM) group at the 5′-end and the AP-site, respectively.

Through immobilized metal affinity chromatography, TeuendoIV was purified to electrophoretic purity, as demonstrated by 15% SDS-PAGE (Figure 1a). The potential AP endonuclease activity was tested using DNA carrying a synthetic AP site, dSpacer. On incubating both ssDNA and dsDNA with the purified TeuendoIV, a 17-nt DNA band, which is the product of the AP endonuclease, was generated (Figure 1b). The cleavage of ssDNA containing a dSpacer is similar to the bacterial EndoIV and human Ape1 [37,38]. At the tested concentration of TeuendoIV, it generated a 16-nt DNA band, indicating that the 3′-exonuclease activity is also possessed by TeuendoIV, which is similar to bacterial EndoIVs [18,19]. Furthermore, the 3′-exonuclease activity of TeuendoIV prefers the dsDNA. To weaken the 3′-exonuclease activity, ssDNAs containing AP site analogues were used as substrate in the major assays for AP endonuclease activity.

After confirmation of AP endonuclease activity, the optimal reaction parameters were determined for TeuendoIV using ssDNA carrying an internal dSpacer as substrate (Figure S1). TeuendoIV showed higher activity at pH values ranging from 7.5 to 9.0, with a maximum activity at pH 8.5. Although EDTA did not inactivate the AP endonuclease, addition of EDTA inhibited the enzymatic activity to some extent, implying the metal ion is necessary for EndoIV. A high concentration of DTT inhibited the AP endonuclease activity. As an enzyme from hyperthermophiles, TeuendoIV showed higher activity at temperatures ranging from 50 to 65 °C.

Divalent metal ions showed different effects on activity (Figure S2). Mg^2+^ promoted AP endonuclease activity, and Mn^2+^ had no clear effect on activity. However, Ni^2+^ and Zn^2+^ showed clear inhibition on activity. In particular, Zn^2+^, which is the preferred cofactor for EcoendoIV [26], is a strong inhibitor of TeuendoIV. Interestingly, TeuendoIV showed clear AP endonuclease activity at the absence of divalent mental ions, implying that it bound some metal ions during recombinant expression in *E. coli* cell.

### 2.2. TeuendoIV Shows AP Endonuclease on Various AP Site Analogues

**Figure 2:**
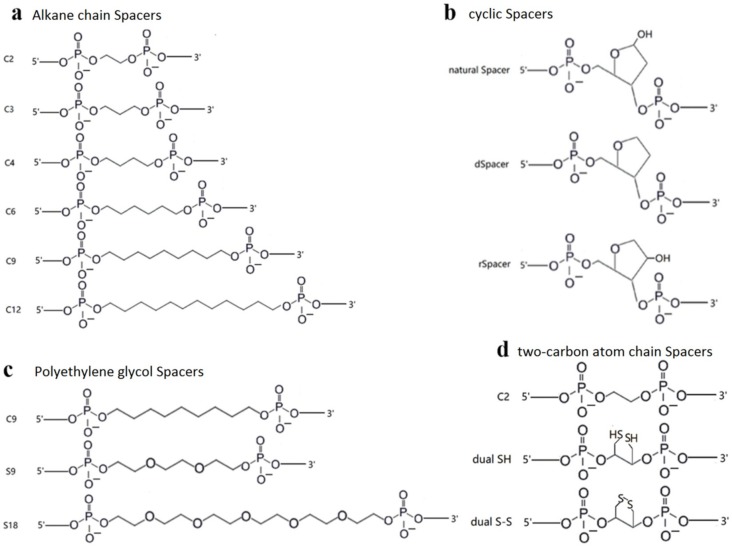
Structures of various AP site analogues. The molecular structure of (**a**) alkane chain Spacers (Spacer Cn), (**b**) cyclic Spacers (natural Spacer, and synthetic dSpacer and rSpacer), (**c**) polyethylene glycol Spacers (Spacer 9 and Spacer 18), and (**d**) two-carbon atom chain Spacers (Spacer C2, Dual SH, and disulfide (S-S)).

**Figure 3:**
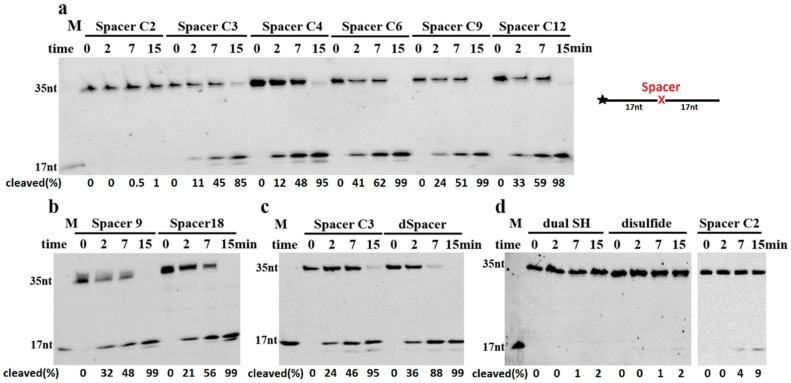
AP endonuclease activity on ssDNAs containing various Spacers. Different ssDNAs (100 nM) containing (**a**) alkane spacers, (**b**) polyethylene glycol spacers, (**c**) dSpacer and spacer C3, and (**d**) sulfur atom spacers were incubated with 5 nM (**a**–**c**) or 10 nM (d) of TeuendoIV at 55 °C for the indicated time. For the Spacer disulfide bond (S-S), the NAD^+^ (1.0 mM) was substituted for DTT in the reaction buffer. Uppercase letter M denotes oligonucleotides marker. The cleavage percentages of substrates are listed at the bottom of each panel. The symbol of black asterisk and the red letter X denote the fluorescein (6-FAM) group at the 5′-end and the AP-sites, respectively.

The natural AP site is not stable under high temperature, so it is generally replaced by several AP site analogues in AP endonuclease activity assays [10]. Various AP site analogues have different molecular structures, which might largely affect the AP endonuclease activity. To understand the cleavage reaction on AP sites, we utilized DNAs containing a range of AP site analogues with different molecular structures (Figure 2) as substrates in cleavage assay. TeuendoIV can hydrolyze the phosphodiester bond 5′ to various alkane chain and polyethylene glycol Spacers with different cleavage efficiency (Figure 3a,b). For alkane chains longer than three carbon atoms, TeuendoIV efficiently cleaved the phosphodiester bond 5′ to the alkane chain (Figure 3a). However, the cleavage efficiency is not proportional to the length of the alkane chain and is almost constant for Spacers longer than three carbon atoms. These results imply that the alkane chains longer than C3 can be efficiently bent out of the DNA backbone to perfectly orient the phosphodiester bond for attacking by the catalytic water molecule. However, under our assay conditions, ssDNA containing an internal Spacer C2 was not cleaved because of the short alkane chain (Figure 3a), implying that the phosphodiester bond 5′ to Spacer C2 is not oriented perfectly into the substrate-binding pocket and cannot be attacked by the coordinated water molecule [26]. For polyethylene glycol Spacers, TeuendoIV hydrolyzed the phosphodiester bond 5′ to Spacers 9 and 18 with a comparable efficiency (Figure 3b). Furthermore, the cleavage efficiencies of Spacer C9 and Spacer 9 were similar (Figure S3), suggesting that the oxygen atoms of polyethylene glycol Spacers have no influence on the cleavage reaction.

The cyclic Spacers have a structure similar to natural AP site deoxyribose, so they might be recognized and cleaved at higher efficiency than linear Spacers. Both Spacer C3 and dSpacer have a three-carbon atom chain between two phosphate groups, but dSpacer has a cyclic deoxyribose-like molecular structure, and Spacer C3 is a linear carbon chain (Figure 2). Our results showed that the AP endonuclease activity of TeuendoIV cleaved the ssDNA containing dSpacer more efficiently than Spacer C3 (Figure 3c). These results indicate that the cyclic larger tetrahydrofuran structure is beneficial to correctly orient the phosphodiester bond 5′ to AP sites into the enzymatic catalytic center for attacking by a water molecule. When the amount of TeuendoIV was increased, ssDNA containing a Spacer C2 was weakly cleaved (Figure 3d). However, the two Spacers Dual SH and disulfide (S-S, the assays were performed in the presence of 1.0 mM NAD^+^), which have the same two-carbon atom chain as Spacer C2, completely blocked the hydrolysis of the phosphodiester bond (Figure 3d). When a five-fold concentration of TeuendoIV was used in the assay, the Spacer C2 was cleaved completely and the Spacer Dual SH was not cleaved (Figure S4). Meanwhile, major DNAs were digested by the 3′-exonuclease activity because of the blockage of AP endonuclease activity by the Spacers C2 and Dual SH.

The kinetic parameters for various AP site analogues (Table 1) show that, except for Spacer C2, the tested AP-site analogues have the comparable K_m_ and k_cat_ values, with a little substrate preference to dSpacer. Although the K_m_ of Spacer C2 is 2-fold higher than those of other AP-site analogues, its k_cat_ show at least 100-fold decrease, resulting in the large decrease of k_cat_/K_m_.

### 2.3. Cleavage of DNA Containing Clustered AP Site Analogues

**Figure 4:**
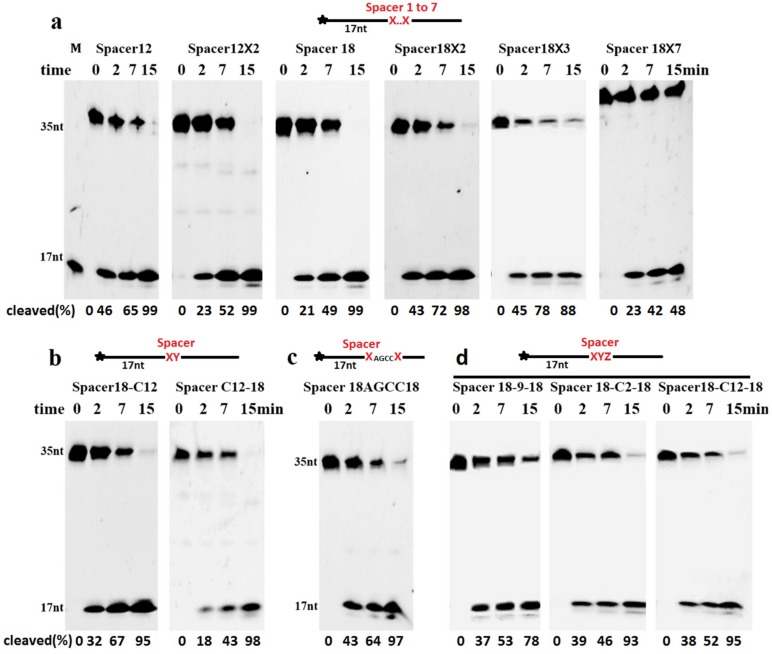
Cleavage of ssDNAs containing clustered AP site analogues. The ssDNAs (100 nM) containing (**a**) consecutive Spacer C12 or 18, (**b**) tandem Spacers C12 and 18, or (**c**) tandem Spacer 18 separated by four normal bases or (**d**) a different middle AP site analogue were incubated with TeuendoIV (5 nM) at 55 °C for the indicated time. The combinations of AP site analogues are listed on the top of each panel. Uppercase letter M denotes oligonucleotides marker. The cleavage percentages of substrates are listed at the bottom of each panel. The symbol of black asterisk and the red letters X, Y and Z denote the fluorescein (6-FAM) group at the 5′-end and the AP-sites, respectively.

Although DNA containing a single Spacer can be cleaved, the clustered AP sites may have effect on cleavage. We performed the cleavage reactions of ssDNAs containing more than one consecutive AP site analogue. Our results showed that TeuendoIV efficiently cleaved the upstream phosphodiester bond of several consecutive AP site analogues, such as Spacer C12 and Spacer 18 (Figure 4a). Even ssDNA containing seven consecutive Spacer 18 damages was cleaved efficiently at the first phosphodiester bond located 5′ to the consecutive AP sites. The DNA containing three clustered cyclic AP sites, dSpacers, also was cleaved with a comparable efficiency to DNA with a single dSpacer (Figure S6). In addition to the consecutive identical AP sites, the phosphodiester bond 5′ to two tandem different AP site analogues was also hydrolyzed efficiently, and the tandem order of two AP site analogues affected the cleavage efficiency to some degree (Figure 4b). If ≥4 normal nucleotides existed between two identical AP sites, the cleavage took place at the 5′-sides of both AP sites (Figure 4c and Figure S7). If the two identical Spacers were separated by another different Spacer, including the Spacer C2, the cleavage site almost was exclusively happened at the phosphodiester bond 5′ to the first AP site (Figure 4d).

We also characterized the cleavage of ssDNAs containing the clustered AP sites analogues that are poorly cleaved when existing alone, such as Spacer C2 and Dual SH. The cleavage efficiency of the phosphodiester bond is proportional to the number of consecutive Spacer C2, even at a lower concentration of TeuendoIV (Figure S8), suggesting that Spacer C2 actually does not inhibit the hydrolysis reaction and only blocks the perfect orientation of the cleaved phosphodiester bond into the enzymatic active center. The perfect orientation became easy when ≥2 Spacer C2 are clustered consecutively. In contrast to a cleavage percentage of 86% for clustered Spacer C2×2, only 12% of the clustered Spacer Dual SH×2 was cleaved even at a 10-fold concentration of enzyme (Figure S8), indicating that the Dual SH group actually inhibits the hydrolysis reaction.

### 2.4. The Base Opposite the AP Site Has Little Effect on dsDNA Cleavage

**Figure 5:**
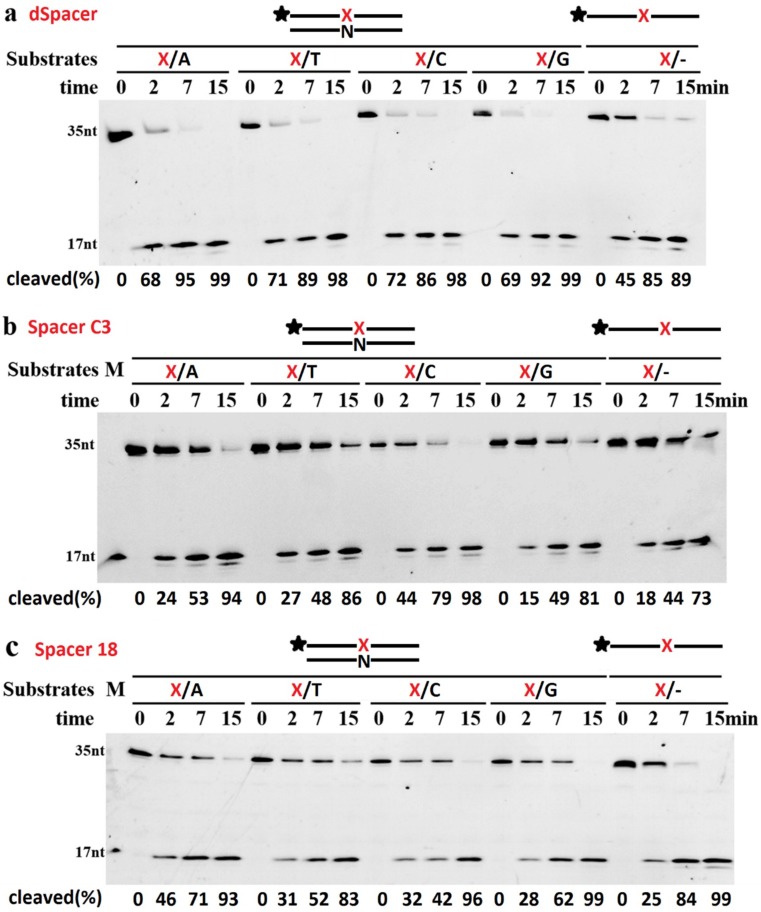
Effect of the base opposite AP site on dsDNA cleavage. TeuendoIV (5 nM) was incubated with 100 nM ssDNA or dsDNAs opposite each of four bases at 55 °C for the indicated time. The Spacers are (**a**) dSpacer, (**b**) Spacer C3, and (**c**) Spacer 18. Uppercase letter M denotes oligonucleotides marker. The cleavage percentages of substrates are listed at the bottom of each panel.

The AP endonuclease activities of TeuendoIV on dsDNA and ssDNA were compared. Generally, TeuendoIV preferred dsDNA to ssDNA for all tested Spacers (Figure 5). The bases (A, T, C, and G) opposite Spacer C3 or 18 had an effect on the dsDNA cleavage reaction in the following order: AP/C > AP/T > AP/A > AP/G (Spacer C3), and AP/A > AP/A > AP/C > AP/T (Spacer 18). However, the AP endonuclease activity of TeuendoIV did not show a clear preference to the base opposite dSpacer.

### 2.5. Cleavage of DNA Containing AP Sites Adjacent to Termini

**Figure 6:**
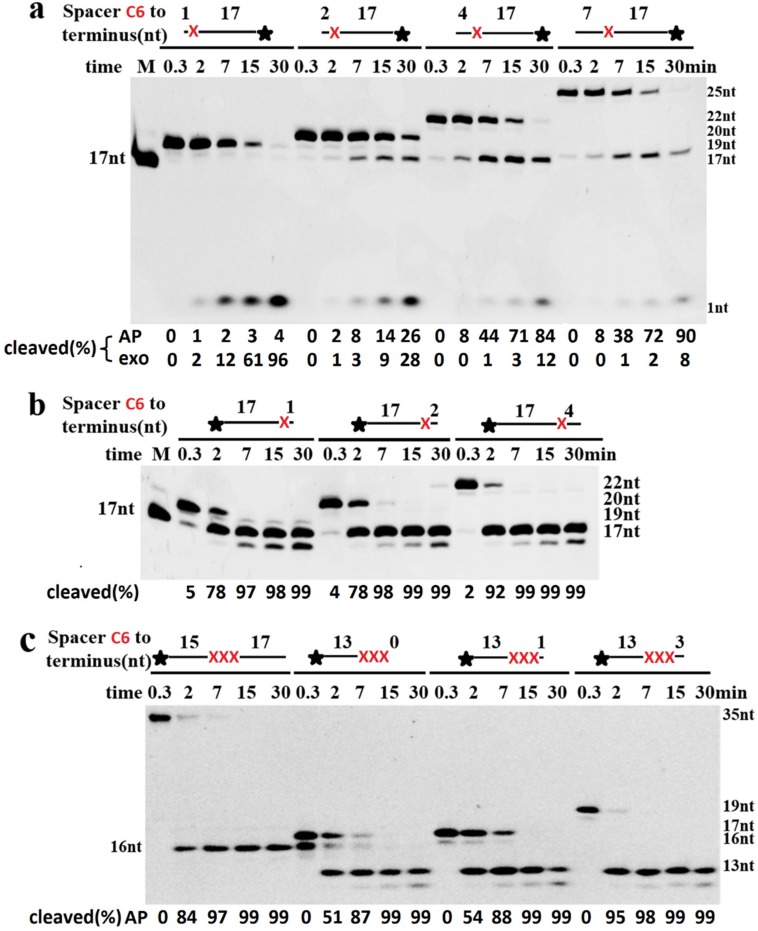
Cleavage of ssDNAs with a Spacer near the 3′-/5′-terminus. TeuendoIV (5 nM) was incubated with 100 nM ssDNA containing one Spacer C6 adjacent to the (**a**) 5′-terminus, (**b**) 3′-terminus, or (**c**) three Spacers (Spacer C6) adjacent to the 3′-terminus at 55 °C for the indicated time. Uppercase letter M denotes oligonucleotides marker. The cleavage percentages of substrates are listed at the bottom of each panel. AP denotes AP endonuclease activity and exo denotes 3′-exonuclease activity. The symbol of black asterisk and the red letter X denote the fluorescein (6-FAM) group at the 5′- or 3’-end and the AP-site, respectively.

The EndoIV has 3′-exonuclease and 3′-repair diesterase activities; the 3′-exonuclease removes normal 3′-nucleotides, and the 3′-repair activity removes abnormal 3′-terminal groups, such as 3′-phosphates, 3′-phosphoglycolates, and 3′-α,β unsaturated aldehydes [11,12]. To cleave the phosphodiester bond 5′ to an AP site, it is generally required that the AP site should be located at the appropriate position of the DNA backbone. For the Spacer adjacent to the 5′-terminus, at least two normal nucleotides were required to be located 5′ to an AP site (Figure 6a). If only one normal nucleotide was placed on the 5′-terminus, almost no product of AP endonuclease was generated. However, the 3′-exonuclease of TeuendoIV was promoted to hydrolyze the 3′-nucleotide, resulting in generation of substantial 3′-FAM mononucleotide (Figure 6a), suggesting that TeuendoIV has strong intrinsic 3′-exonuclease similar to bacterial EndoIV [18,19]. For the AP sites adjacent to the 3′-terminus, the existence of additional normal 3′-nucleotides is not necessary for hydrolyzing the phosphodiester bond 5′ to the AP site (Figure 6b). However, the normal 3′-nucleotides showed promotion on the AP endonuclease activity. When the single Spacer C6 in Figure 6b was changed to three consecutive clustered ones, the hydrolysis model did not change, predominantly hydrolyzing the phosphodiester bond located at the 5′ side of the first Spacer (Figure 6c). Furthermore, if more than one Spacer C6 was located at the 3′-terminus, the hydrolysis reaction also took place at the first 3′-terminal phosphodiester bond, i.e., the one between two 3′-terminal Spacer C6 (Figure 6c, second substrate, 0.3 min).

### 2.6. Extension by DNA Polymerase Requires the Removal of 3′-Terminal AP Site Analogues

**Figure 7:**
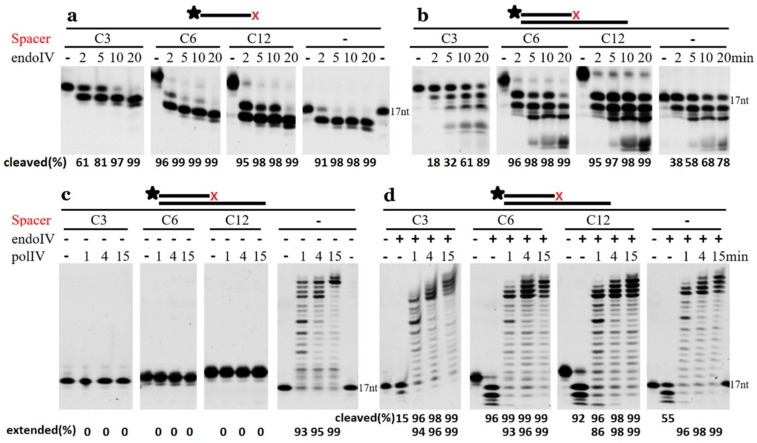
Extension by DNA polymerase after removal of 3′-terminal AP site analogues. (**a**) ssDNA and (**b**–**d**) 3′-recessive dsDNA with a 3′-terminal Spacer (Spacer C3, C6, or C12) or a normal nucleotide were incubated with 10 nM TeuendoIV alone (**a**,**b**) at 55 °C for the indicated time. The 3′-recessive dsDNA were incubated with *Sulfolobus acidocaldarius* DNA polymerase IV at 50 °C for the indicated time in the (**c**) absence or (**d**) presence of 10 nM TeuendoIV. After removing terminal AP sites with TeuendoIV at 50 °C for 5 min (**d**), the DNA polymerase was added into reaction mixtures, and then an additional incubation was performed. The cleavage and extension percentages of substrates are listed at the bottom of each panel. The symbol of black asterisk and the red letter X denote the fluorescein (6-FAM) group at the 5′-end and the AP-sites, respectively.

Spacers C3, C6, and C12 can be removed from the 3′-terminus of ssDNAs in the order of Spacer C6 > C12 > normal base > C3, and normal nucleotides are further removed after the 3′-Spacers (Figure 7a). The dsDNAs with 3′-recessive Spacers took a similar hydrolysis model as ssDNAs (Figure 7b) and generated the 3′-OH. If the 3′-Spacers were not removed by TeuendoIV, it blocked the polymerization reaction by DNA polymerase IV that lacks 3′-exonuclease activity (Figure 7c). After removing the 3′-blocked Spacers by TeuendoIV, *Sulfolobus acidocaldarius* DNA polymerase IV can extend the recessive primer strand using normal dNTP (Figure 7d).

### 2.7. Key Residues for Recognition and Cleavage of Phosphodiester Bonds

**Figure 8:**
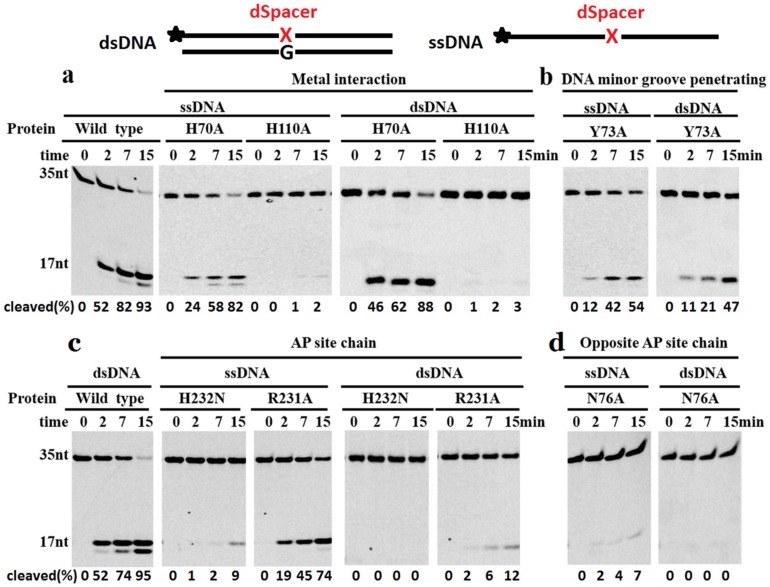
Cleavage reactions of wt (wild type) and mutant TeuendoIV. Mutations on key conserved residues potentially involved in (**a**) coordinating metal ion cofactors, (**b**) penetrating into the duplex minor groove, and binding the (**c**) AP site-containing strand and (**d**) complementary strand were used to assay AP endonuclease activities (5 nM enzymes) on ssDNA and dsDNA with an internal dSpacer (100 nM) at 55 °C for the indicated time. The cleavage percentages of substrates are listed at the bottom of each panel. The symbol of black asterisk and the red letter X denote the fluorescein (6-FAM) group at the 5′-end and the AP-site, respectively.

To analyze the catalytic mechanism of TeuendoIV, a series of site mutations were made on its conserved key residues. These site mutations are divided into four groups, including metal ion coordination mutations H70A and H110A, DNA minor groove penetration mutation Y73A, AP site-binding mutations R231A and H232N, and complementary strand-binding mutation N76A. Consistent with the results of PfuendoIV [36], mutation H70A did not result in complete inactivation of TeuendoIV (Figure 8a). Since H110A completely inactivated TeuendoIV, H110 might play a more important role than H70 in coordinating the metal ion cofactor. The Y73A mutation only weakly decreased the enzymatic activity (Figure 8b), indicating that the penetration of residue Y73 into the DNA minor groove contributes minor role during recognition of AP sites. Based on the co-crystal structure of EcoendoIV and dsDNA containing an AP site, several residues are responsible for binding the AP site-containing strand and normal complementary strand, respectively [26,27,28]. The effects of residue mutations on binding AP site-containing strand are interesting (Figure 8c). H232N almost completely lost the AP endonuclease activity on ssDNA and dsDNA. In addition to binding the 5′-side base of the AP site, the residue H231 in EcoendoIV (corresponding to the residue H232 in TeuendoIV) also coordinates the divalent metal ions [26]. Therefore, the possibility is that H232A strongly inactivate the AP endonuclease activity of TeuendoIV because of its inability to coordinate metal ion cofactor. In contrast to H232A, R231A selectively inactivated the AP endonuclease activity on dsDNA, suggesting that the residue R231 only participates in the recognition of AP sites in dsDNA. Considering that N76 is only involved in binding the complementary strand [26,27], it is plausible that the N76A obtained minor activity on ssDNA and completely lost the activity on dsDNA (Figure 8d), indicating that the disruption of binding the complementary strand might cause serious defects in recognizing and binding the AP site in ssDNA.

### 2.8. Structure Comparison of TeuendoIV and EcoendoIV

**Figure 9:**
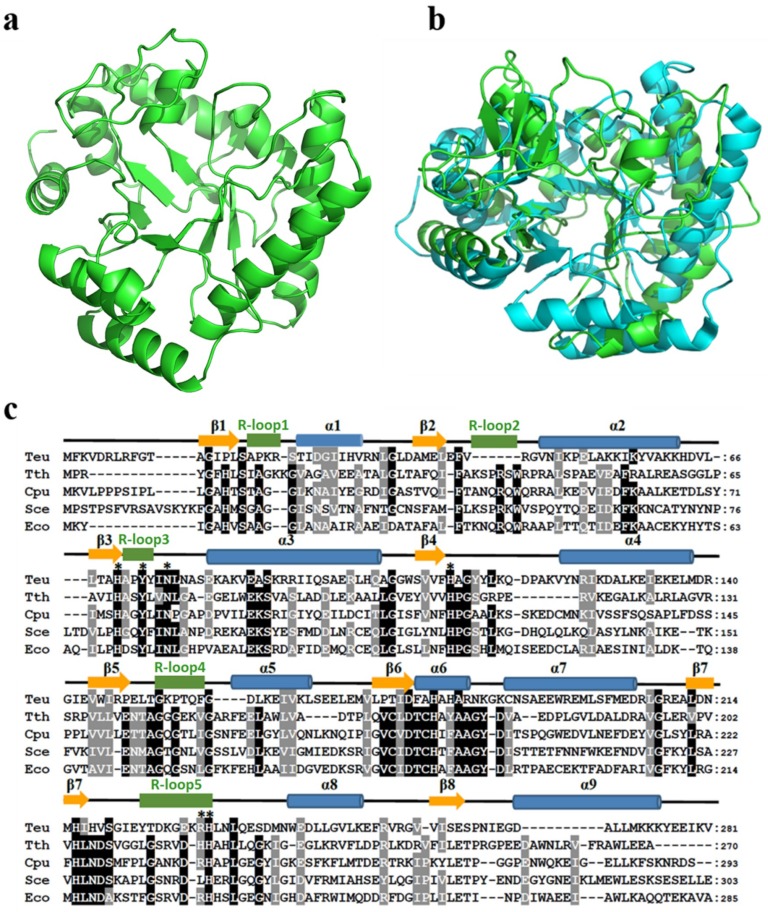
Homology model of TeuendoIV and multi-alignment of EndoIVs. (**a**) Modeled structure of TeuendoIV. (**b**) Superimposition of TeuendoIV (green) and EcoendoIV (cyan). (**c**) Multiple sequence alignment of EndoIVs. The EndoIVs are from *T. eurythermalis*, *T. thermophilus* (PDB ID: 3AAM), *Chlamydophila pneumoniae*, *S. cerevisiae* and *E. coli* (PDB ID: 1QTW). The secondary structures of EcoendoIV are shown at the top of sequences. Cylinders indicate α-helices, arrows indicate β-strands, and five green rectangles indicate DNA-binding recognition loops 1–5. Identical and similar residues are shaded in black and gray, respectively. The mutated residues are marked by asterisks.

Because no crystal structure of archaeal EndoIV has been solved, a modeled topology of TeuendoIV was built based on the bacterial EcoendoIV crystal structure (Figure 9a), which is composed of nine α-helixes, five DNA-binding recognition loops (R-loop), and eight β-strands [26]. Compared with EcoendoIV, TeuendoIV has a more disordered N-terminal domain and lacks an R-loop2 (Figure 9).

Archaeal TeuendoIV shows very weak sequence similarity to these from bacteria and *S. cerevisiae* (Figure 9c). The sequence identity between TeuendoIV and EcoendoIV is just 24% (Figure 9c), implying that there are differences between their topologies. The modeled structure of TeuendoIV also supported this speculation (Figure 9a,b). TeuendoIV lacks a short peptide, the R-loop2 in bacterial and *S. cerevisiae* EndoIV. In the crystal structure of EcoendoIV and dsDNA, this R-loop2 has much interactions with DNA, including deoxyriboses, bases and phosphates, and might play important roles in hydrolyzing the phosphodiester bonds 5′ to the AP site [26,27]. The absence of R-loop2 did not result in enzymatic inactivity, but the removal of N-terminal 21 residues inactivated the TeuendoIV [39], suggesting that the N-terminal secondary structures β1 and R-loop1 are required for catalytic activity. The phylogenic tree also shows that TeuendoIV branches from bacterial and eukaryotic EndoIV (Figure S9).

## 3. Materials and Methods

### 3.1. Materials

KOD-plus DNA polymerase was purchased from Toyobo (Osaka, Japan). Expression vector pET28a and *E. coli* Rosetta 2(DE3)pLysS were purchased from Merck (Darmstadt, Germany). The Ni-NTA resin was bought from Bio-Rad (Hercules, CA, USA). The Spacer phosphoramidites were purchased from Glen Research (Sterling, VA, USA) and ChemGenes (Wilmington, MA, USA). Oligonucleotides were synthesized in Biosune (Shanghai, China). All the other chemicals and reagents were of analytical grade.

### 3.2. Construction of Expression Plasmids

*T. eurythermalis* A501 was cultured at 85 °C, and its genomic DNA was extracted by phenol-chloroform and precipitated using isopropyl alcohol [35]. The gene encoding TeuendoIV was amplified from the genomic DNA using a pair of specific primers (Table S1) by PCR and inserted into pET28a between the *Nde* I and *Bam*H I sites, producing a recombinant expression vector, pET28a-TeuEndoIV. The site-directed mutations were constructed on the basis of the TeuendoIV expression plasmid according to the protocol of the QuikChange^®^ Site-Directed Mutagenesis Kit from Agilent (Santa Clara, CA, USA) using respective primers (Table S1). The base sequences of inserted genes were confirmed by DNA sequencing.

### 3.3. Expression and Purification of TeuendoIV

Various expression plasmids were transformed into *E. coli* Rosetta 2(DE3)pLysS to express recombinant EndoIV and its mutants. Isopropyl-1-thio-b-D-galactopyranoside (IPTG) was added into bacterial cultures (OD_600_ ≈ 0.8) with a 0.5 mM final concentration to induce the expression of target proteins for 20 h at 16 °C. Bacterial pellets were resuspended with ice-cold lysis buffer (20 mM Tris-HCl pH 7.9, 300 mM NaCl, 10 mM imidazole, 5 mM β-mercaptoethanol (β-ME), 1 mM phenylmethylsulfonyl fluoride (PMSF) and 10% glycerol) for breaking cells by sonication. Lysates were clarified by centrifugation at 16,000× *g* for 30 min at 4 °C. Then the supernatants were purified by the immobilized Ni^2+^ affinity chromatography. After loading the supernatant onto a column pre-equilibrated with lysis buffer, the resin was washed with 100 column volumes of lysis buffer containing 20 mM imidazole. Finally, the target proteins were eluted with lysis buffer containing 200 mM imidazole. The purity of eluted fractions was confirmed by 15% SDS-PAGE. The purified proteins were stored in small aliquots at −20 °C

### 3.4. Activity Assay of TeuendoIV

The base sequences of oligodeoxyribonucleotides used in the activity assay are listed in Table S2. The AP site analogues, dSpacer, alkane chain (Spacers C2, C3, C4, C6, C9 and C12), polyethylene glycol (Spacers 9 and 18), Dual SH, and disulfide bond (S-S) were introduced into oligonucleotides that were used as substrates to determine the effect of AP site structure on DNA cleavage reaction. The disulfide bond (S-S) was introduced by oxidizing the dual SH by NAD^+^. The strands carrying the AP site analogues were fluorescently labeled with FAM at the 5′-end. The dsDNAs with different base pairs (N/AP, N denotes one of A, T, C, G) were used to characterize the effects of the base opposite the AP site analogues on AP endonuclease activity. To prepare dsDNA substrates, the FAM-labeled strand was annealed with unlabeled complementary strand in a mole ratio of 1:1.5 by boiling for 5 min at 95 °C and slowly cooling down to room temperature.

AP endonuclease activity was determined as described with some modification [12]. Standard reaction solutions contained 100 nM 5′-FAM-labeled AP site-containing ssDNA or dsDNA and the specified amount of TeuendoIV in buffer consisting of 20 mM Tris-HCl pH 7.6, 100 mM NaCl, and 0.2 mM EDTA. After optimization of reaction buffer, all reactions were performed at 55 °C in buffer consisting of 20 mM Tris-HCl pH 7.5, 50 mM NaCl, 1 mM MgCl_2_, 0.5 mM DTT, and 0.5 mM EDTA. For the DNA substrate containing a disulfide bond Spacer, the NAD^+^ (1.0 mM) was substituted for DTT in the reaction buffer. The exonuclease activity of TeuendoIV and DNA polymerase activity of *Sulfolobus acidocaldarius* DNA polymerase IV were performed in buffer consisting of 20 mM Tris-HCl pH 8.8, 10 mM KCl, 10 mM (NH_4_)_2_SO_4_, 2 mM MgSO_4_, 100 ng/mL BSA and 0.1% TritonX-100. The reactions were incubated at 55 °C for the indicated time and were stopped by adding an equal volume of loading buffer (50 mM EDTA, 8 M urea, 0.2% SDS, 0.1% bromophenol blue, 0.1% xylene cyan) to reaction samples. Then, the reaction products were analyzed by electrophoresis in 15% polyacrylamide gels (8 M urea). After electrophoresis, the gels were imaged using an FL9500 fluorescent scanner and quantified with the analysis software (GE Healthcare, Chicago, IL, USA).

To quantitatively compare the cleavage efficiency between various AP site analogues, the kinetic parameters (K_m_ and k_cat_) of TeuendoIV on ssDNAs containing various AP-site analogues were calculated using double reciprocal plotting. The 5′-FAM-labelled ssDNAs containing Spacer C3, C4, C6, C12, 9, 18 and dSpacer (0.05, 0.1, 0.25, 0.5, 1.0, 2.0 and 5.0 μM) were incubated with 25 nM TeuendoIV for 5 min. For cleavage reaction of Spacer C2, 500 nM TeuendoIV was incubated with the above concentration of ssDNA for 30 min. The cleavage products were quantitated and used to calculate the initial reaction rates, which were used to calculate the values of K_m_ and k_cat_.

### 3.5. Multiple Sequence Alignment and Constructing Phylogenetic Tree

The multiple sequence alignment was performed using MUSCLE (Multiple Sequence Comparison by Log Expectation) on the website of http://www.ebi.ac.uk/Tools/msa/muscle/. The phylogenetic tree was built by MEGA using sequences from archaea and bacteria (Table S3).

### 3.6. Structure Modeling of EndoIV

The modeled structure of TeuendoIV was constructed using ProMod3 on the SWISS-MODEL server (https://www.swissmodel.expasy.org/) [40]. The crystal structure of EcoendoIV (PDB ID: 1QTW) was used as the template during homologous modeling. The two structures of EcoendoIV and TeuendoIV were aligned using Pymol (Schrodinger LLC, New York, NY, USA). The PyMOL Molecular Graphics System, version 1.5.0.3).

## 4. Discussion

Bacterial and archaeal EndoIV can hydrolyze natural AP site [12,36]. We confirm that archaeal EndoIV can also hydrolyze various AP site analogues with different molecular structures. The length of the AP site is an important factor in the hydrolysis efficiency of the phosphodiester bond 5′ to an AP site analogue. Our results show that the length of the AP site should be longer than two carbon atoms for efficient hydrolysis, and too short an alkane chain (Spacer C2) cannot be efficiently treated by TeuendoIV (Figure 3a). The molecular conformation is another determinant of cleavage efficiency. The dSpacer, which has a molecular structure similar to the natural AP site, is preferably recognized and cleaved than Spacer C3, implying that the circular ribose-like structure is a promoter to hydrolyze the phosphodiester bond 5′ to dSpacer. Compared with the weak cleavage of phosphodiester bond 5′ to Spacer C2, two Spacers, dual thiol and disulfide bond structures, completely block the cleavage reaction, implying that, in addition to the short length, the groups of the disulfide bond and dual thiol also cause hindrance for orienting and/or attacking the 5′-side phosphodiester bond by water molecule.

The position of an AP site in DNA also determines the efficiency of the hydrolysis reaction. When the AP site analogues are located near the 5′-terminus, at least two normal 5′-nucleotides are required for the efficient hydrolysis of phosphodiester bonds. However, the 3′-terminal AP site analogues, even several clustered Spacers, can be removed efficiently, implying that both the AP endonuclease and 3′-exonuclease activities of EndoIV might use a similar substrate-binding and hydrolysis mechanism. For the clustered farther 3′-Spacers, the cleavage reaction can take place at the first 3′-terminal phosphodiester bond (Figure 6c, second substrate, 0.3 min), indicating the 3′-exonuclease of TeuendoIV can remove the first Spacer of the farther 3′-terminal consecutive Spacers.

During hydrolysis of phosphodiester bonds by AP endonucleases, the DNA backbone is bound by several conserved residues [26,27,28]. The EcoendoIV-DNA interactions are mainly provided by residues R230 and H231 for binding the AP site, residue N75 for binding the complementary strand, and residue Y72 DNA for penetrating into the minor groove. These conserved residues correspond to R231 and H232, N76, and Y73 in TeuendoIV. Residue Y72 in EcoendoIV plays a central role in recognizing the AP site, and the mutation Y72A results in a 1000-fold decrease in activity [27]. Similarly, the mutation Y73A leads to a strong loss of activity of TeuendoIV (Figure 8b), indicating that R-loop3 plays a similar role in recognizing the AP site. The mutations on residues for binding the AP site strand should decrease the cleavage reaction of dsDNA and ssDNA, and our results confirm their harmful effects on the AP endonuclease activity of TeuendoIV (Figure 8c). It is conceivable that the mutations on residues for binding the AP site-containing strand are harmful to cleaving both ssDNA and dsDNA. However, the R231A mutation only seriously decreases the activity on the dsDNA and has a minor harmful effect on the cleavage of ssDNA. The mutations on the residues for binding the complementary strand are thought to be destructive for the hydrolysis of phosphodiester bonds in dsDNA, not those in ssDNA. Actually, the N76A mutation completely inactivates the AP endonuclease activity on dsDNA and leads to partial activity on ssDNA (Figure 8d).

Although R-loop2 in EcoendoIV also provides interactions with the DNA backbone and bases, the residues corresponding to R-loop2 are absent or largely changed in TeuendoIV (Figure 9c). When the N-terminal 21 residues are deleted, the truncated TeuendoIV loses its AP endonuclease activity [39], indicating the R-loop1 and R-loop2 are important for binding DNA. These results might be interpreted by the complex crystal structure of TeuendoIV and dsDNA or ssDNA. Therefore, to compare the differences between bacterial and archaeal EndoIV in their structural and biochemical properties, solving the crystal structure of TeuendoIV will be important.

EndoIV possesses both AP endonuclease and 3′-phosphodiesterase activities, both of which might use the same water molecule to attack the phosphodiester bonds, i.e., the same catalysis mechanism for two enzymatic activities. The AP endonuclease requires EndoIV to interact with the DNA backbone upstream and downstream of an AP site, whereas the 3′-phosphodiesterase (3′-repair diesterase/3′-exonuclease) cannot interact with the backbone downstream of an AP site, which does not exist for the 3′-terminal Spacer. Therefore, the mutations on residues that interact with the 3′ backbone of an internal AP site should have little effect on the hydrolysis of the 3′-terminal phosphodiester bond by 3′-exonuclease, as well as the removal of 3′-terminal AP sites or 3′-blocking groups by AP endonuclease/3′-repair diesterase.

Compared with ExoIII, EndoIV has more homologues in archaea, and even more than one homologue can exist in one species. For example, two and three EndoIV homologues are present in the *P. furiosus* and *Methanothermobacter thermautotrophicus* genomes, respectively [36]. Among these EndoIV homologues, generally only one possesses AP endonuclease and 3′-exonuclease activities [36]. *M. thermautotrophicus* also has a homologue of ExoIII, which does not possess the AP endonuclease activity but functions as an endonuclease specific to dU damage [41]. Unlike the usually coupled AP endonuclease/3′-repair diesterase/3′-exonuclease, in the human pathogen *Neisseria meningitides* these activities are endowed to two separated homologues [42]. Meanwhile, the lower level of sequence identity between TeuendoIV and EcoendoIV and the phylogenetic tree of EndoIV (Figure S9) imply a farther ancestral relationship between archaea and bacteria EndoIV.

Depurination/depyrimidination occurs more frequently at high temperatures [5]. Hence, hyperthermophiles face a serious spontaneous mutation resulting from AP sites and dU damage [5,34]. The BER pathway, as the main AP site repair pathway, is important for tolerating high temperature and avoiding mutagenesis in archaea. *T. eurythermalis* does not encode ExoIII, implying EndoIV plays an important role in the BER pathway. Considering that bacterial EndoIV possesses RNA-specific 3′-exonuclease activity [43,44,45] and human Ape1 can process abasic and oxidized ribonucleotides embedded in DNA [46], the TeuendoIV might function in the repair of non-AP site-type damage and RNA metabolism.

## Figures and Tables

**Table 1:** Kinetic parameters K_m_ and k_cat_ of TeuendoIV for AP-site analogues.

Substrates	K_m_ (μM)	k_cat_ (min^−1^)	k_cat_/K_m_ (min^−1^·μM^−1^)
dSpacer	0.35 ± 0.03	0.59 ± 0.05	1.68 ± 0.15
Spacer C2	0.16 ± 0.02	0.0021 ± 0.0002	0.013 ± 0.002
Spacer C3	0.37 ± 0.03	0.23 ± 0.02	0.62 ± 0.06
Spacer C4	0.41 ± 0.04	0.33 ± 0.03	0.80 ± 0.08
Spacer C6	0.36 ± 0.03	0.37 ± 0.04	1.03 ± 0.10
Spacer C12	0.41 ± 0.04	0.35 ± 0.03	0.85 ± 0.08
Spacer 9	0.34 ± 0.03	0.36 ± 0.03	1.06 ± 0.10
Spacer 18	0.37 ± 0.04	0.42 ± 0.04	1.14 ± 0.11

K_m_ and k_cat_ for cleaving ssDNAs containing dSpacer and Spacer C3, C4, C6, C12, 9, 18 were calculated by double reciprocal plotting using the initial reaction rates at various substrate concentrations (0.05, 0.1, 0.2, 0.5, 1.0, 2.0 and 5.0 μM) and 25 nM TeuendoIV where incubation time is 5 min. Considering that the very low activity of TeuendoIV on Spacer C2, the concentration of TeuendoIV and the incubation time were increased to 500 nM and 30 min, respectively. The cleaved products are less than 10% (Table S4), which indicate that the kinetic experiments were performed during periods when initial rates hold. The initial reaction rates (Table S4) were used to calculate the values of K_m_ and k_cat_, and the graphs of the double reciprocal plotting are shown in Figure S5. All data are the means of three independent experiments.

## Supplementary Materials

Supplementary materials can be found at http://www.mdpi.com/1422-0067/20/1/69/s1.
