# Supplementary material for: Thermococcus Eurythermalis Endonuclease IV Can Cleave Various Apurinic/Apyrimidinic Site Analogues in ssDNA and dsDNA
Source: Int J Mol Sci. 2018 Dec 24;20(1):69. doi: 10.3390/ijms20010069 (PMC6341776; doi:10.3390/ijms20010069)
Supplement: Supplementary file 1 [file ijms-20-00069-s001.pdf]

***Thermococcus eurythermalis* endonuclease IV can cleave various apurinic/apyrimidinic site analogues in ssDNA and dsDNA**

Wei-Wei Wang [1,†], Huan Zhou [2,†], Juan-Juan Xie [1], Gang-Shun Yi [1], Jian-Hua He [2], Feng-Ping Wang [1,3], Xiang Xiao [1,3] and Xi-Peng Liu [1,3,*]

[1] State Key Laboratory of Microbial Metabolism, School of Life Sciences and Biotechnology, Shanghai Jiao Tong University, 800 Dong-Chuan Road, Shanghai 200240, China; www1037554814@sjtu.edu.cn (W.W.W.); purplexjj@163.com (J.J.X.); 13166228531@163.com (G.S.Y.); fengpingw@sjtu.edu.cn (F.P.W.); zjxiao2018@sjtu.edu.cn (X.X.)

[2] Shanghai Institute of Applied Physics, Chinese Academy of Sciences, No. 239 Zhangheng Road, Shanghai 201204, China; zhouhuan@sinap.ac.cn (H.Z.); hejianhua@sinap.ac.cn (J.H.H.)

[3] State Key Laboratory of Ocean Engineering, School of Naval Architecture, Ocean and Civil Engineering, Shanghai Jiao Tong University, 800 Dong-Chuan Road, Shanghai 200240, China

* Correspondence: xpliu@sjtu.edu.cn

† These authors contributed equally to this work.

Supplementary materials

Supplementary Figures

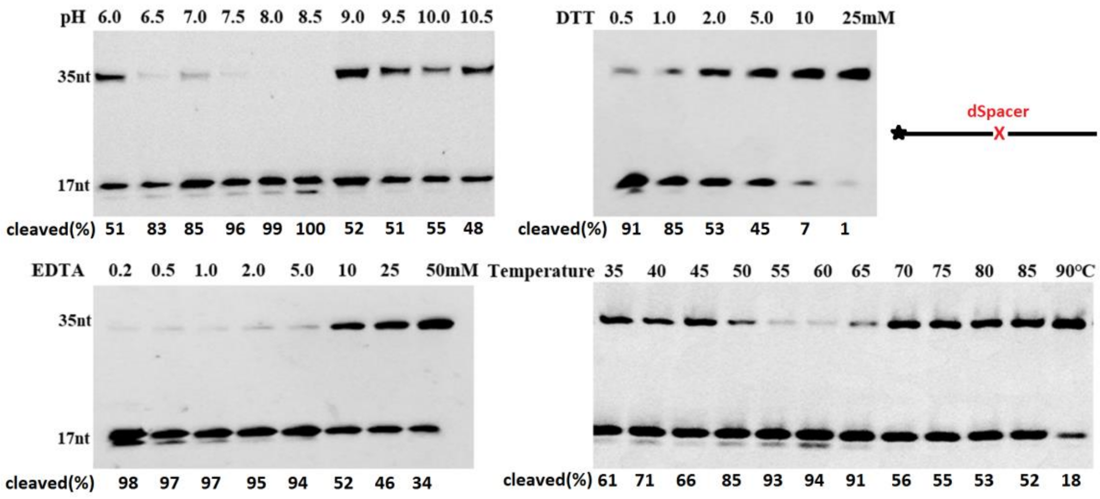

Figure S1 Optimization of reaction conditions. The pH value, concentrations of EDTA and DTT, and reaction temperature were sequentially optimized. The cleavage percentages of substrates are listed at the bottom of each panel.

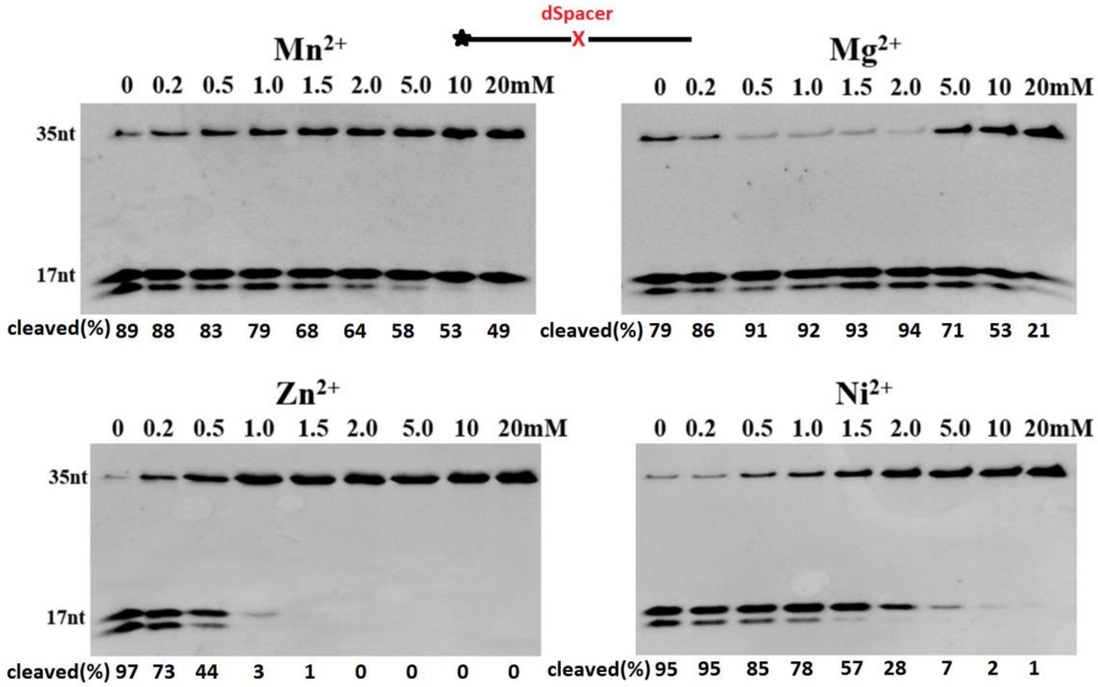

Figure S2 Optimization of divalent metal ions. Different concentrations of Zn^{2+}, Mg^{2+}, Ni^{2+}, and Mn^{2+} were included in the optimized reaction buffer for the assays of AP endonuclease activity. The cleavage percentages of substrates are listed at the bottom of each panel.

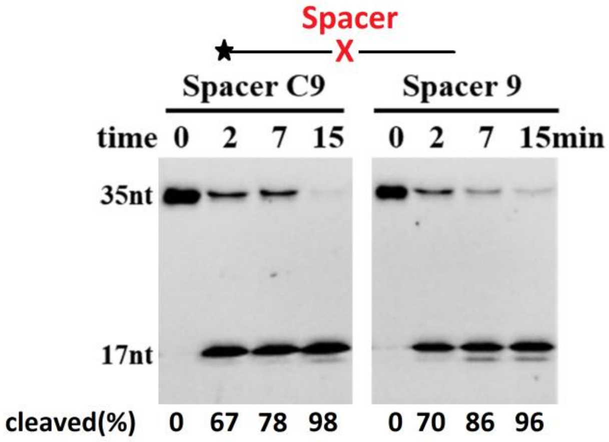

Figure S3 Cleavage of ssDNAs containing Spacer C9 or 9. SsDNAs (100 nM) containing an internal Spacer C9 or Spacer 9 were incubated with TeuendoIV (5 nM) at 55°C for the indicated time in the optimized reaction buffer. The cleavage percentages of substrates are listed at the bottom of each panel.

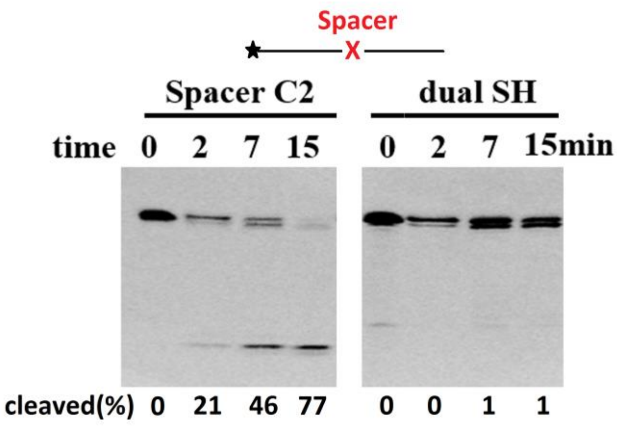

Figure S4 Cleavage of ssDNAs containing single Spacer C2 or Dual SH. SsDNAs (100 nM) containing one Spacer C2 or Dual SH were incubated with TeuendoIV (50 nM) at 55°C for the indicated time. The cleavage percentages of substrates are listed at the bottom of each panel.

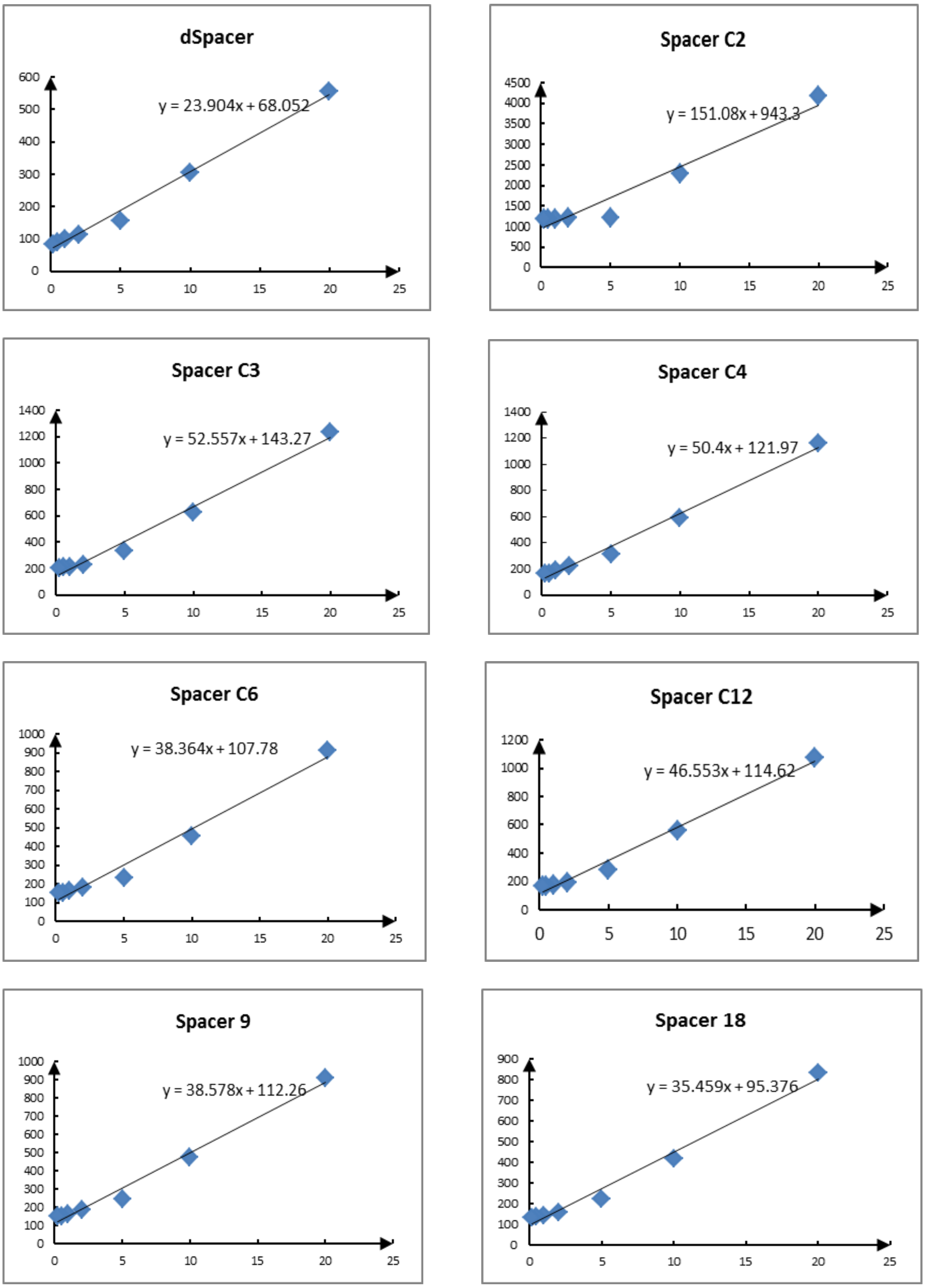

Fig. S5 Graphs of the double reciprocal plotting of various AP-site analogues.

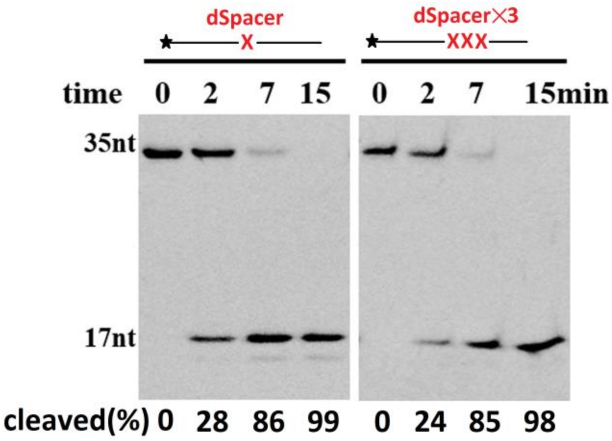

Figure S6 Cleavage of ssDNAs containing one or three clustered dSpacers. SsDNAs (100 nM) containing one or three internal dSpacers were incubated with TeuendoIV (5 nM) at 55°C for the indicated time in the optimized reaction buffer. The cleavage percentages of substrates are listed at the bottom of each panel.

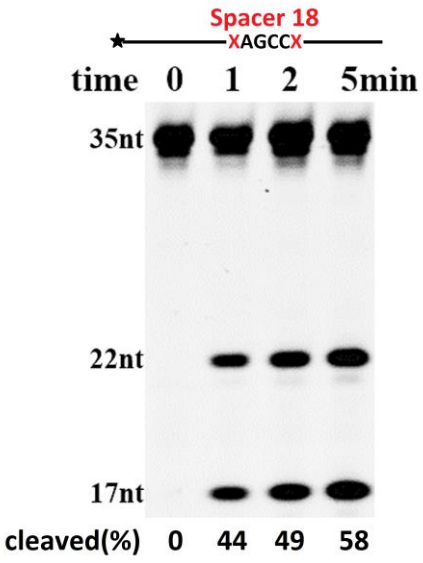

Figure S7 Cleavage of ssDNA containing two Spacer 18s separated by four normal nucleotides. The ssDNAs (100 nM) were incubated with TeuendoIV (2 nM) at 55°C for the indicated time in the optimized reaction buffer. The cleavage percentages of substrates are listed at the bottom of each panel.

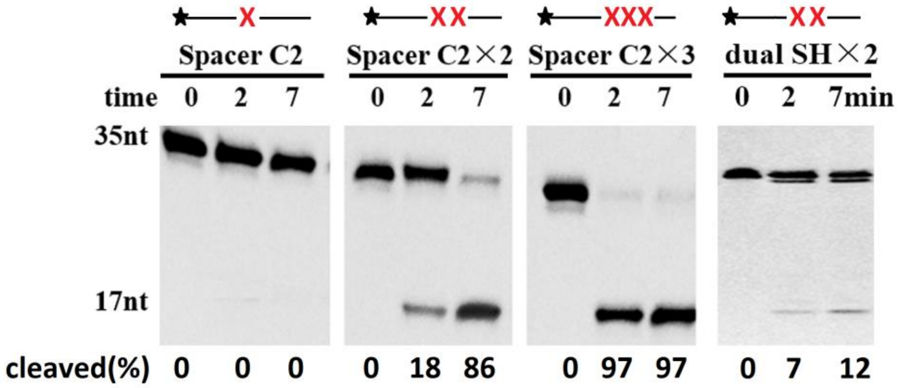

Figure S8 Cleavage of ssDNAs containing one or clustered Spacer C2 and dual SH. SsDNAs (100 nM) containing 1-3 internal Spacer C2s or dual SH were incubated with TeuendoIV (5 nM for Spacer C2 and 50 nM for Spacer dual SH) at 55°C for the indicated time. The cleavage percentages of substrates are listed at the bottom of each panel.

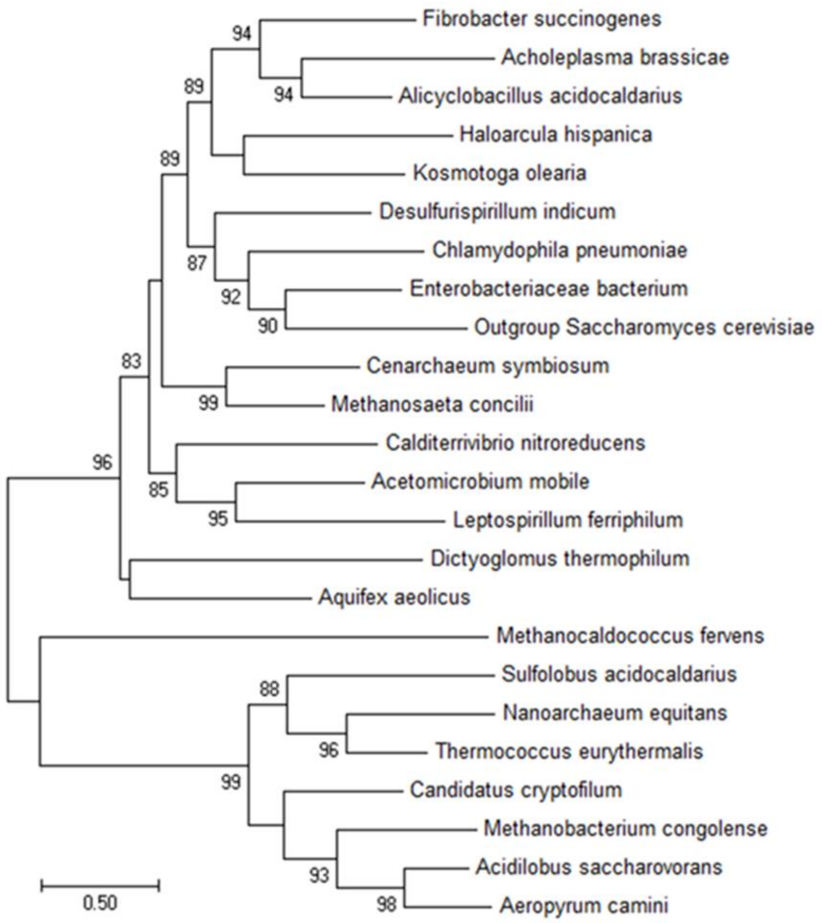

Figure S9 Phylogenetic tree of EndoIV. Selected EndoIV from typical bacteria and archaea were used to construct the phylogenetic tree. The bootstrap values are listed at the branch.

Supplementary Tables

Table S1 Oligonucleotides used for constructing plasmids

Oligos	Sequences (5' – 3') [a]	Comments
TeuendoIV-F	tgaaa**catatg**aaagtacttcctcctc	TeuEndoIV
TeuendoIV-R	ttgcttcg**ggatcc**aaaag cac	
H70A-F	ctgct cacagctGCc gcgccctact acat	H70A
H70A-R	agtagggcgc gGCagctgtg agcaggacg	
Y73A-F	tcac gcgcccGCct acatcaacct caacg	Y73A
Y73A-R	g aggttgatgt agGCgggcgc gtgagctg	
N76A-F	ccctact acatcGCcct caacgcgagc ga	N76A
N76A-R	tcgcgttg aggGCgatgt agtagggcgc g	
H110A-F	agcgt cgttttcGCc gccggctact acct	H110A
H110A-R	agtagccggc gGCgaaaacg acgctccag	
R231A-F	a gggcgagaag GCgcacctga acctccag	R231A
R231A-R	aggt tcaggtgcGC cttctcgccc ttatc	
H232N-F	gcgagaag aggGCcctga acctccagga g	H232N
H232N-R	tggaggt tcaggGCcct cttctcgccc tt	

[a] The changed bases are shown in uppercase letters.

Table S2 Oligonucleotides used for analyzing enzymatic activity

Sequences (5′-3′) [a]	Damages	Figures
*CAGCCAGGTGTCTCACTXAGCCGACTCGCCACAGT	X=dU	1b
*CAGCCAGGTGTCTCACTXAGCCGACTCGCCACAGT	X=Spacer C2	3,4,5, S1-3,S4,S6-7
*CAGCCAGGTGTCTCACTXAGCCGACTCGCCACAGT	X=Spacer C3	
*CAGCCAGGTGTCTCACTXAGCCGACTCGCCACAGT	X=Spacer C4	
*CAGCCAGGTGTCTCACTXAGCCGACTCGCCACAGT	X=Spacer C6	
*CAGCCAGGTGTCTCACTXAGCCGACTCGCCACAGT	X=Spacer C9	
*CAGCCAGGTGTCTCACTXAGCCGACTCGCCACAGT	X=Spacer C12	
*CAGCCAGGTGTCTCACTXAGCCGACTCGCCACAGT	X=dSpacer	
*CAGCCAGGTGTCTCACTXAGCCGACTCGCCACAGT	X=Spacer 9	
*CAGCCAGGTGTCTCACTXAGCCGACTCGCCACAGT	X=Spacer 18	
*CAGCCAGGTGTCTCACTXAGCCGACTCGCCACAGT	X=Dual SH	
*CAGCCAGGTGTCTCACTXAGCCGACTCGCCACAGT	X=S-S	
*CAGCCAGGTGTCTCACTXXGCCGACTCGCCACAGT	X=Spacer C12X2	4, S5
*CAGCCAGGTGTCTCACTXXGCCGACTCGCCACAGT	X=Spacer 18X2	
*CAGCCAGGTGTCTCACTXXXCCGACTCGCCACAGT	X=Spacer 18X3	
*CAGCCAGGTGTCTCACTXXXXXXXCTCGCCACAGT	X=Spacer 18X7	
*CAGCCAGGTGTCTCACTXYGCCGACTCGCCACAGT	X=Spacer 18, Y=Spacer C12	
*CAGCCAGGTGTCTCACTYXGCCGACTCGCCACAGT	X=Spacer 18, Y=Spacer C12	
*CAGCCAGGTGTCTCACTXAGCCXACTCGCCACAGT	X=Spacer 18	
*CAGCCAGGTGTCTCACTXYXCCGACTCGCCACAGT	X=Spacer 18, Y=Spacer C12	
*CAGCCAGGTGTCTCACTXYXCCGACTCGCCACAGT	X=Spacer 18,Y=Spacer 9	
*CAGCCAGGTGTCTCACTXYXCCGACTCGCCACAGT	X=Spacer 18,Y=Spacer C2	
*CAGCCAGGTGTCTCACTXXXCCGACTCGCCACAGT	X=dSpacer	S4
*CAGCCAGGTGTCTCACTXXGCCGACTCGCCACAGT	X=Spacer C2	S6
*CAGCCAGGTGTCTCACTXXXCCGACTCGCCACAGT	X=Spacer C2	
*CAGCCAGGTGTCTCACTXXGCCGACTCGCCACAGT	X=Dual SH	S7
TXAGCCGACTCGCCACAGT*	X=Spacer C6	6
CTXAGCCGACTCGCCACAGT*	X=Spacer C6	
CACTXAGCCGACTCGCCACAGT*	X=Spacer C6	
TCTCACTXAGCCGACTCGCCACAGT*	X=Spacer C6	
*CAGCCAGGTGTCTCACTXA	X=Spacer C6	
*CAGCCAGGTGTCTCACTXAG	X=Spacer C6	
*CAGCCAGGTGTCTCACTXAGCC	X=Spacer C6	
*CAGCCAGGTGTCTCAXXXAGCCGACTCGCCACAGT	X=Spacer C6	
*CAGGTGTCTCACTXXX	X=Spacer C6	
*CAGGTGTCTCACTXXXC	X=Spacer C6	
*CAGGTGTCTCACTXXXCCG	X=Spacer C6	
*CAGCCAGGTGTCTCACTX	X=Spacer C3	7
*CAGCCAGGTGTCTCACTX	X=Spacer C6	
*CAGCCAGGTGTCTCACTX	X=Spacer C12	

*CAGCCAGGTGTCTCACT		
ACTGTGGCGAGTCGGCTAAGTGAGACACCTGGCTG	complementary strand	2b,5,7,8
ACTGTGGCGAGTCGGCTTAGTGAGACACCTGGCTG	complementary strand	
ACTGTGGCGAGTCGGCTCAGTGAGACACCTGGCTG	complementary strand	
ACTGTGGCGAGTCGGCTGAGTGAGACACCTGGCTG	complementary strand	

[a] Asterisks denote the fluorescein (6-FAM) group at the 5′- or 3′-end. The damaged bases (AP site analogues or dU) are denoted by the letters X and Y in the sequences and are annotated in the "Damage" column. The damage-containing strands are shown in blue with the Spacer damage in red, and the complementary strands are shown in black.

Table S3 EndoIV used for constructing phylogenetic tree

Strain name	Protein Name	Gene locus	Lineage
Acidilobus saccharovorans	Endonuclease IV	ASAC_1468	Archaea
Aeropyrum camini	Endonuclease IV	ACAM_1316	Archaea
Korarchaeum cryptofilum	Endonuclease IV	Kcr_0075	Archaea
Cenarchaeum symbiosum	Endonuclease IV	CENSYa_1925	Archaea
Haloarcula hispanica	Endonuclease IV	HAH_2520	Archaea
Methanobacterium congolense	Putative endonuclease 4	MCBB_0201	Archaea
Methanocaldococcus fervens	Putative endonuclease 4	Mefer_1095	Archaea
Methanosaeta concilii	Apurinic-apyrimidinic endonuclease	MCON_1877	Archaea
Nanoarchaeum equitans	NEQ077a	NEQ077a	Archaea
Sulfolobus acidocaldarius	Endonuclease IV	Saci_0015	Archaea
Fibrobacter succinogenes	Apurinic endonuclease Apn1	FSU_2936	Bacteria
Acetomicrobium mobile	Apurinic endonuclease APN1	Anamo_0665	Bacteria
Acholeplasma brassicae	Endodeoxyribonuclease IV	BN85311130	Bacteria
Alicyclobacillus acidocaldarius	Deoxyribonuclease IV	Aaci_1017	Bacteria
Aquifex aeolicus	Endonuclease IV	Aq_1629	Bacteria
Calditerrivibrio nitroreducens	Deoxyribonuclease IV	Calni_1384	Bacteria
Chlamydophila pneumoniae	Endonuclease IV	CP_0014	Bacteria
Desulfurispirillum indicum	Apurinic endonuclease Apn1	Selin_1546	Bacteria
Dictyoglomus thermophilum	Endonuclease IV	DICTH_1988	Bacteria
Enterobacteriaceae bacterium	Endonuclease IV	F652_1793	Bacteria
Kosmotoga olearia	Apurinic endonuclease Apn1	Kole_0273	Bacteria
Leptospirillum ferriphilum	Apurinic endonuclease	LFML04_0897	Bacteria
Saccharomyces cerevisiae	APN2	YBL019W	Eukaryota

Table S4 The maximum values of product amount and the initial rates during the incubation period

Substrate concentration (μM)		0.05	0.1	0.2	0.5	1.0	2.0	5.0
dSpacer	Cleaved substrate (%)	18.0	16.5	16.0	8.9	5.1	2.8	1.2
	Initial rate (μM/min)	0.0018	0.0033	0.0064	0.0089	0.01	0.011	0.012
Spacer C2	Cleaved substrate (%)	14.0	13.2	12.2	4.9	2.5	1.3	0.5
	Initial rate (μM/min)	0.00023	0.00044	0.00081	0.00082	0.00083	0.00087	0.00083
Spacer C3	Cleaved substrate (%)	8.1	8.1	7.3	4.4	2.4	1.2	0.5
	Initial rate (μM/min)	0.00081	0.0016	0.003	0.0044	0.0048	0.0048	0.005
Spacer C4	Cleaved substrate (%)	8.6	8.4	8.0	4.5	2.7	1.5	0.6
	Initial rate (μM/min)	0.00086	0.0017	0.0032	0.0045	0.0054	0.006	0.006
Spacer C6	Cleaved substrate (%)	11.1	11.0	10.6	5.6	3.1	1.6	0.7
	Initial rate (μM/min)	0.0011	0.0022	0.0043	0.0056	0.0062	0.0065	0.0067
Spacer C12	Cleaved substrate (%)	9.3	9.2	9.2	5.2	2.9	1.5	0.6
	Initial rate (μM/min)	0.00093	0.0018	0.0036	0.0052	0.0058	0.0061	0.006
Spacer 9	Cleaved substrate (%)	11.0	10.5	10.2	5.4	3.1	1.6	0.7
	Initial rate (μM/min)	0.0011	0.0021	0.0041	0.0054	0.0062	0.0065	0.0067
Spacer 18	Cleaved substrate (%)	11.9	11.8	11.3	6.4	3.5	1.8	0.8
	Initial rate (μM/min)	0.0012	0.0024	0.0045	0.0064	0.007	0.0073	0.0076

The percentage of substrate converted to product during the incubation period, and the initial rates at the increasing substrate concentrations of various AP-site analogues are listed.
